# Bayesian risk assessment model of human cryptosporidiosis cases following consumption of raw Eastern oysters (*Crassostrea virginica*) contaminated with *Cryptosporidium* oocysts in the Hillsborough River system in Prince Edward Island, Canada

**DOI:** 10.1016/j.fawpar.2020.e00079

**Published:** 2020-03-19

**Authors:** Thitiwan Patanasatienkul, Spencer J. Greenwood, J.T. McClure, Jeff Davidson, Ian Gardner, Javier Sanchez

**Affiliations:** a Department of Health Management, Atlantic Veterinary College, University of Prince Edward Island, PEI, Canada; b Department of Biomedical Sciences, Atlantic Veterinary College, University of Prince Edward Island, PEI, Canada

**Keywords:** Risk assessment, *Cryptosporidium*, Bayesian inference, Human infection, Oyster

## Abstract

*Cryptosporidium* spp. has been associated with foodborne infectious disease outbreaks; however, it is unclear to what extent raw oyster consumption poses a risk to public health. Control of *Cryptosporidium* in shellfish harvest seawater in Canada is not mandatory and, despite relay/depuration processes, the parasite can remain viable in oysters for at least a month (depending on initial loads and seawater characteristics). Risks of human infection and illness from exposure to oysters contaminated with *Cryptosporidium* oocysts were assessed in a Bayesian framework. Two data sets were used: counts of oocysts in oysters harvested in Approved, Restricted, and Prohibited zones of the Hillsborough River system; and oocyst elimination rate from oysters exposed to oocysts in laboratory experiments. A total of 20 scenarios were assessed according to number of oysters consumed in a single serving (1, 10 and 30) and different relay times. The median probability of infection and developing cryptosporidiosis (e.g. illness) due to the consumption of raw oysters in Prince Edward Island was zero for all scenarios. However, the 95th percentiles ranged from 2% to 81% and from 1% to 59% for probability of infection and illness, respectively. When relay times were extended from 14 to 30 days and 10 oysters were consumed in one serving from the Restricted zones, these probabilities were reduced from 35% to 16% and from 15% to 7%, respectively. The 14-day relay period established by Canadian authorities for harvesting in Restricted zones seems prudent, though insufficient, as this relay period has been shown to be enough to eliminate fecal coliforms but not *Cryptosporidium* oocysts, which can remain viable in the oyster for over a month. Extending relay periods of 14 and 21 days for oysters harvested in Restricted zones to 30 days is likely insufficient to substantially decrease the probability of infection and illness. The highest risk was found for oysters that originated in Prohibited zones. Our findings suggest that *Cryptosporidium* oocysts are a potential cause of foodborne infection and illness when consuming raw oysters from Hillsborough River, one of the most important oyster production bays on Prince Edward Island. We discuss data gaps and limitations of this work in order to identify future research that can be used to reduce the uncertainties in predicted risks.

## Highlights

• Risk of infection and illness of cryptosporidiosis in humans by consuming raw oysters from PEI is likely to be negligible.
• Depuration time of 14 days might not be enough to reduce *Cryptosporidium* oocysts contamination in oysters in bays of PEI.
• More field data need to be obtained to reduce uncertainties in predicted risks.

## 1 Introduction

Cryptosporidiosis is one of the greatest concerns in food production worldwide. Some of the *Cryptosporidium* spp. are human enteric pathogens that inflict considerable morbidity on people and can cause mortality in immunosuppressed individuals (Graczyk et al., 2006). They are considered one of the most important enteropathogens worldwide, due to the increasing incidence in industrialized countries and the large numbers of children and immunocompromised individuals affected in developing countries (Shirley et al., 2012), and because the illnesses caused by these parasites range from mild gastroenteritis to life-threatening syndromes (King and Monis, 2007). Transmission is via environmentally-resistant *Cryptosporidium* oocysts, which are infectious when excreted. *Cryptosporidium* oocysts are transmissible to humans and other animals via direct contact with contaminated fecal material or by contact with, or consumption of, contaminated sources such as water and food (Dixon et al., 2011); however, not every *Cryptosporidium* spp. found in the wild pose risk to human health. The species of primary concern to human health are *C. parvum* and *C. hominis* (Isaza et al., 2015).

Oysters filter large volumes of seawater and, during this process, they can accumulate and concentrate marine toxins (NSW, 2011), chemical contaminants (de Mora et al., 2005), bacteria, viruses, and parasites, such as *Cryptosporidium* oocysts (Iwamoto et al., 2010; Willis et al., 2013b), increasing the potential risk of infection for humans upon consumption of raw or undercooked shellfish (Downey and Graczyk, 2007; Robertson, 2007; Smith and Nichols, 2010). While the epidemiological importance of contamination of oysters with *Cryptosporidium* spp. remains unknown as a cause of foodborne cryptosporidiosis (Budu-Amoako et al., 2011), data reported in the medical literature on infectious gastrointestinal illness (IGI) likely represent a small portion of actual cases (approximately 160–610 actual cases per reported case), and it is widely accepted that the number of foodborne illnesses are globally under-reported (MacDougall et al., 2008; Majowicz et al., 2005; Robertson, 2007). In Prince Edward Island (PEI), Canada, 1–11 cases of cryptosporidiosis are reported to local health authorities every year (Department of Health and Wellness, 2016); however, between 160 and 6700 cases could be reasonably expected to occur annually, based on the above studies, if the risk is similar across Canada.

In Canada, Fisheries and Oceans Canada (DFO) (CFIA-EC-DFO, 2015; DFO, 2012) defines Approved, Restricted, and Prohibited zones for oyster fishing. An “Approved” zone is open for oyster fishing from August 15th to October 31st; oysters harvested during this period do not require depuration and can go directly to market. A “Restricted” zone is fished from May 1st to July 15th, and all harvested oysters are placed in depuration or relay stations in clean waters outside of the Restricted zone to clear the oysters of monitored contaminants (only chemical and fecal coliform levels are tested for) for at least 14 days (CFIA-EC-DFO, 2013). No oysters can be fished for consumption from “Prohibited zones,” as these zones are contaminated with wastewater release sites as, for example, in the municipalities of Charlottetown and Stratford, including the regional hospital in PEI, Canada. Oysters are very effective in depurating *E. coli* (Formiga-Cruz et al., 2003), but their ability to depurate *Cryptosporidium* oocysts is limited (Graczyk et al., 2006). In shellfish, static depuration apparently has minimal ability to remove oocysts from tissue, with oocysts still being detected after 22–37 days (Willis et al., 2013b). Depuration has been found to be ineffective at inactivating viable *Cryptosporidium* oocysts, as 50% of *Cryptosporidium* oocysts that received 8–14 days of depuration were still viable (Gómez-Couso et al., 2003).

Under the *Safe Food of Canadians Act* (S.C. 2012, c. 24), it is mandatory to control the levels of fecal coliforms and chemicals in Canadian seawater; however, there is no legislation requiring public health officials to test for *Cryptosporidium* oocysts in potentially contaminated shellfish or water (CFIA, 2019; Minister of Justice, 2019). Since *Cryptosporidium* spp. have been detected in various wild animal species (Ryan et al., 2014), these wildlife could have passed oocysts to oysters in an open sea. Additionally, the presence of *Cryptosporidium* oocysts in oysters could be due to sewage discharged into harvest areas, illegal harvest of oysters from sewage-contaminated waters, sewage runoff after heavy rains or flooding (Iwamoto et al., 2010), and agricultural contaminant runoff (Schijven et al., 2004). Studies on the above contamination sources have shown no relationship between fecal coliforms or *E. coli* levels and concentrations of *Cryptosporidium* oocysts in water samples (Willis et al., 2013b). In addition, there is no association between shellfish contaminated with *Cryptosporidium* oocysts and contamination with other coliform bacteria (Gómez-Couso et al., 2003). Thus, fecal coliform counts alone are not reliable indicators of water quality in shellfish harvesting areas, as has been reported (Rippey, 1994).

For this study, a Bayesian risk assessment model was developed to assess different scenarios in relation to the probability of developing infection and gastrointestinal illness (e.g. risk of illness) associated with consumption of raw oysters contaminated with *Cryptosporidium* oocysts in PEI. We hypothesized that this probability remains high even after 14 and 30 days of depuration of oysters harvested in contaminated zones (e.g. Restricted), where water standard control procedures are based only on fecal coliform counts. Findings from the present study will provide evidence to support the establishment of regulatory controls for event-based monitoring and to assess the need to close or reopen shell-fishing zones in PEI.

## 2 Materials and methods

### 2.1 Bayesian framework and modules

**Fig. 1:**
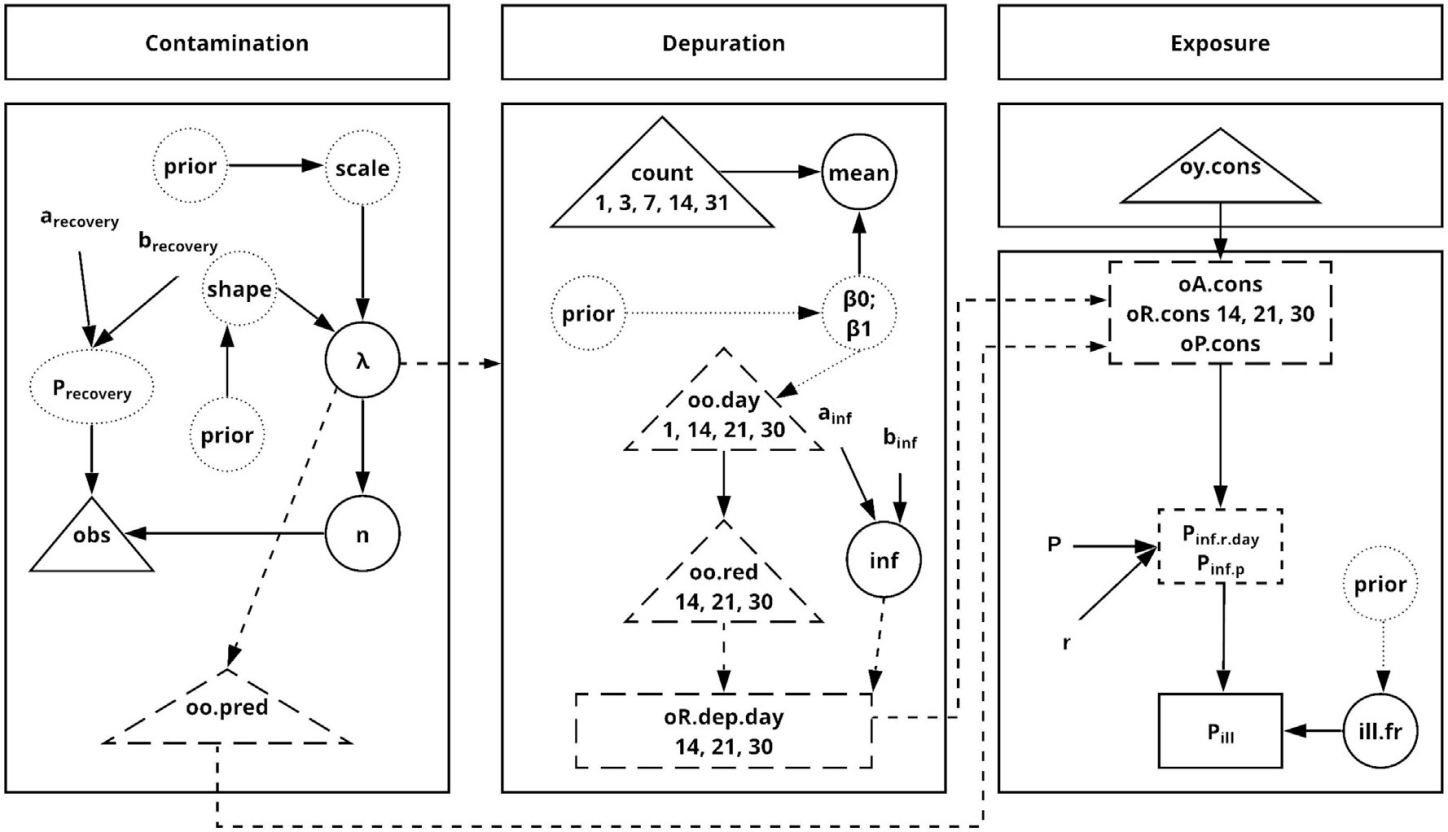
Graphic description of the core model. Data are indicated by triangles; circles and ovals represent stochastic nodes (random variables and priors); rectangles are variables estimated by equations. Dashed lines are predicted variables. See Table 2 for description of parameters, parents, and distributions or relationships.

A Bayesian approach was used to model the individual risk of illness attributable to the consumption of raw oysters contaminated with *Cryptosporidium* spp. oocysts. Fig. 1 depicts a schematic view of the risk assessment model, which is divided into three modules: 1) oyster contamination (number of oocysts/oyster), 2) depuration, and 3) exposure, which involves raw oyster consumption, number of oocyst in a serving, dose-response model, and infection and illness. The outcomes of interest from each module are described in Table 1. All variables used in these modules, and input parameters, probability distributions, fixed values, and equations are summarized in Table 2.

**Table 1:** Description of the risk assessment modules used to estimate the probability of infection and illness of human cryptosporidiosis after consumption of raw oysters in Prince Edward Island.

Module	Outcomes	Definition
Oyster contamination	*oo.pred*	Number of oocysts in oysters
Oyster depuration	*oR.dep14*, *oR.dep21*, and *oR.dep30*	Number of oocysts after 14, 21, and 30 days of relay for oysters from Restricted zones only
Exposure	*oR.cons14*, *oR.cons21*, *oR.cons30*, and *oP.cons*	Number of viable oocysts consumed per person per serving size (1, 10, and 30) from oysters from Restricted zones according to the 3 relay times (14, 21, and 30) and Prohibited zones
• Dose-response	P	Meta-analysis regression parameters using a fractional Poisson model with information from the literature
• Infection	P_inf_	Probability of infection given consumed dose using the dose-response model parameters for oysters from Restricted and Prohibited zones
• Illness	P_ill_	Probability of illness given infection for oysters from Restricted and Prohibited zones

**Table 2:** Description of parameters, parents, and distributions or relationships used in the modules of the *Cryptosporidium* spp. risk assessment.

	Description	Variable/parameter	Parent(s)	Distribution/relationship
Contamination	Concentration of oocysts in an oyster	λ	Shape, scale	Gamma(shape, scale)
	Shape parameter gamma distribution	Shape	Prior	Gamma(0.5, 10^−4^)
	Scale parameter gamma distribution	Scale	Prior	Gamma(0.5, 10^−4^)
	Number of oocysts in an oyster	n	λ	Poisson(λ)
	Number of oocysts detected in an oyster	obs	P_recovery_, n	Binomial(P_recovery,_ n)
	Recovery efficiency	P_recovery_	a_recovery_, b_recovery_	Beta(a_recovery_, b_recovery_)
	Shape and scale parameter for recovery efficiency	a_recovery_, b_recovery_	Constant	3.322, 3.322
Depuration	Oocyst counts in oysters in lab depuration trial (at days 1, 3, 7, 14, and 31)	Count	–	Poisson(count)
	Average of counts on day of measurement	β_0_; β_1_	Count	=exp(β_0_ + β_1_ ∗ day)
	Coefficient of Poisson model	β_0_; β_1_	Prior	Normal(0, 10^−4^)
	Predicted number of oocysts after depuration	oo.day	–	=exp(β_0_ + β_1_ ∗ day)
	Oocysts reduction rate (reduction in the number of oocysts at days 14, 21, and 30)	oo.red.day	oo.1, oo.day	=((oo.1 − oo.day)/oo.1)
	Number of viable oocysts in Restricted zone	oR.dep.day	λ, oo.red.day, inf	=λ ∗ (1 − oo.red.day) ∗ inf
	Probability of an oocyst remaining viable	inf	a_inf_, b_inf_	Beta(a_inf_, b_inf_)
	Shape parameter for viable probability	a_inf_	Constant	#infected oocyst + 1 = 194
	Shape parameter for viable probability	b_inf_	Constant	#oocyst − #infected oocyst + 1 = 3366
	Raw oyster consumption	oy.cons	Constant	1, 10 and 30
Exposure	Ingested level of *Cryptosporidium* (dose = D)	oA.cons; oR.cons.day; oP.cons	oy.cons, oR.dep.day	=(oy.cons ∗ oR.dep.day) ∗ 0.77 0.77 = proportion of oocysts post-shuck
	Probability of infection given ingested dose	P_inf.r.day_; P_inf.p_	P, r, D	=P ∗ (1 − e^(−r∗D)^)
	Pathogen-specific constant for dose-response model	r	Constant	0.018
	Fraction of susceptible host in dose-response model (set to 1 for exponential model)	P	Constant	1
	Probability of an infected subject becoming ill	P_ill_	ill.fr	Uniform(ill.fr_min_, ill.fr_max_)
	Fraction of the infected subjects who become ill	ill.fr	Prior	Min = 0.2, max = 0.7

#### 2.1.1 Oyster contamination module

**Fig. 2:**
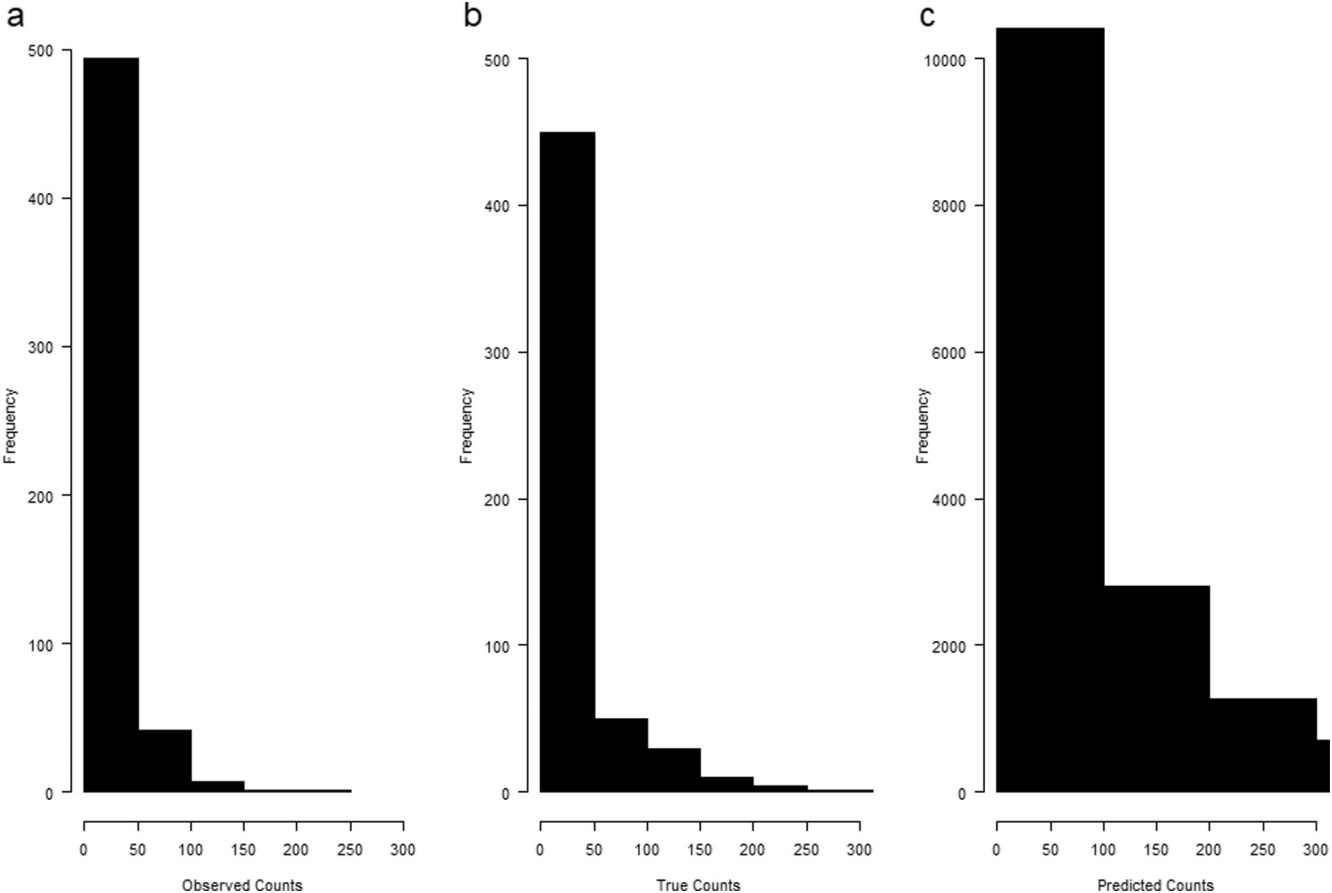
Observed (a), true (b), and predicted (c) *Cryptosporidium* oocyst counts from the hierarchical model. True counts refer to the number of oocysts after adjusting for analytical recovery. Predicted counts refer to posterior predictive values of the true count including both variability in n among oysters, variability in lambda among oysters and parametric uncertainty in the scale and shape parameters.

The numbers of *Cryptosporidium* oocysts per oyster in Approved (*A*), Restricted (*R*), and Prohibited (*P*) zones were provided by. (Willis et al., 2013a) and are depicted in Fig. 2a. Briefly, oocysts/oyster were counted according to protocols previously published by Willis et al. (2013b) and Aguirre et al. (2016), in approximately 650 *Crassostrea virginica* (shell length, 73–100 mm) collected from the three zones in the Hillsborough River system of Charlottetown (Prince Edward Island, Canada) between July 2011 and July 2012. The study has shown that oocysts from oysters harvested in Restricted zones can be contaminated at similar loads as oysters from Prohibited zones, so we assumed similar parasite loads for all oysters harvested from these zones, except for, oysters harvested in Approved zones that were assumed to have zero contamination (Willis et al., 2013a).

We followed the approach outlined by Schmidt et al. (2013), Schmidt and Emelko (2011), and Emelko et al. (2010) to model the number of oocysts per oyster. Briefly, a hierarchical model was fit to account for three types of random variability: 1) random sampling error, 2) analytical error, and 3) non-constant analytical recovery. This model additionally assumes that the concentration of oocysts among oysters followed a gamma distribution, and so the true number of oocysts in an oyster would follow a Poisson-gamma distribution (random sampling error). The analytical error is the difference between the number of oocysts detected in a sample (observed counts) and the number of oocysts actually present in the oyster (true counts), estimated by the enumeration method. If each oocyst has a probability of being observed (e.g., analytical recovery), then the number of oocysts is binomially distributed. The recovery efficiency was assumed non-constant and was modelled using a beta distribution.

The number of oocysts/oyster detected is affected by the recovery efficiency of the extraction technique. Analytical recovery efficiency was incorporated using the approach suggested by Schmidt et al. (2013), where the number of observed oocysts in an oyster followed a binomial distribution, conditional on the actual number of oocysts and the analytical recovery (P_recovery_) of the enumeration method, where P_recovery_ varies randomly according to a beta distribution with parameters a_recovery_ and b_recovery_*.* We estimated the two shape parameters of the beta distribution to both be 3.32 assuming median recovery proportions of 50% and a 97.5th percentile of 84% based on the unpublished data (J. McClure and S. Greenwood, Personal communication, November 30, 2019).

Table 1, Table 2 show the hierarchical structure of the probabilistic model and description of the parameters. The following equations describe the hierarchical model, where *i* is an index variable for sampled oysters:

(1) $$\lambda_{i}\sim \text{Gamma}\left(\text{shape},\text{scale}\right)$$

(2) $$n_{i}\sim \text{Poisson}\left(\lambda_{i}\right)$$

(3) $${\mathrm{obs}}_{i}\sim \text{Binomial}\left(P_{\text{recovery}\_i},n_{i}\right)$$

(4) $$P_{\text{recovery}\_i}\sim \text{Beta}\left(a_{\text{recovery}},b_{\text{recovery}}\right)$$

The parameters of the gamma distribution were then used to predict the number of oocysts per oyster (*oo.pred*, Table 1) to assess our scenario simulations.

#### 2.1.2 Depuration module

Experimental data on *Cryptosporidium* oocyst depuration by commercial *Crassostrea virginica* (shell length of 73–100 mm) were used to model the number of oocysts/oyster at different depuration times. Oysters were exposed for one day in tanks containing 1000 oocysts/1 L of Instant Ocean artificial seawater at a salinity of either 12 or 28 ppt. After exposure, oysters were transferred to a clean static tank system every day until 7 days post-exposure and subsamples of 15 oysters were removed at 1, 3, 7, 14, and 31 days post-exposure for enumeration of oocyst counts (variates called *oo.day*: *oo.1*, *oo.3*, *oo.7*, *oo.14*, and *oo.31*, respectively; see Table 2).

The relationship between the number of oocysts/oyster and depuration time was estimated using a Poisson model. We used coefficients from this model to predict the number of oocysts remaining after depuration at days 14, 21, and 30 (*oo.14*, *oo.21*, and *oo.30*). Our model predicted mean oocyst counts ranging from 39 to 26 and 16 at days 14, 21, and 30, respectively (data not shown). The Canadian Shellfish Sanitation Program (CSSP) Manual of Operations, which (Chapter 2) states that controlled relaying and depuration of oysters harvested in conditionally restricted or restricted zones must be done for at least 14 days, unless a company has undergone a specific verification by the Canadian Food Inspection Agency (CFIA) (CFIA-EC-DFO, 2015). The CSSP also establishes that relaying (transfer from conditionally restricted or restricted zones) to an uncontaminated, approved water source shellfish from 14 to 21 days must be analysed for fecal coliforms with a minimum of one sample. In addition, shellfish relayed in excess of 21 days may be exempt from the testing requirement, at the discretion of CFIA (CFIA-EC-DFO, 2015). The 30-day relay period is part of the scenarios explained in the following sections. It was chosen to assess the residual contamination at 30 days of relay because these periods have been shown to be enough to eliminate fecal coliforms but not *Cryptosporidium* oocysts (Gómez-Couso et al., 2003; Schijven et al., 2013), which can remain viable after filtration periods of 7–33 days or more and still be infectious to humans (Freire-Santos et al., 2000; Robertson, 2007; Willis et al., 2014).

The depuration count data and Poisson regression were used to assess the depuration rate, and estimate the depuration reduction rates at days 14 (*oo.red.14*), 21 (*oo.red.21*), and 30 (*oo.red.30*), as presented in Table 2.

Two factors were considered during depuration. First, we assumed that only oysters harvested in Restricted zones undergo a depuration process. Oysters from Prohibited zones are harvested illegally (only for personal consumption, and represents less than 1% of consumed raw oysters) and, hence, are neither relayed nor commercialized. Second, during the depuration or relay process the infectivity of oocysts decreases because not every oocyst retains the ability to excyst and produce human infection. The level of contamination in a single oyster harvested in a Restricted zone (number of oocysts/oyster) was estimated at days 14 (*oR.dep.14*), 21 (*oR.dep.21*), and 30 (*oR.dep.30*) after depuration, taking into account depuration reduction rates, infectivity rates, and the contamination of a single oyster harvested in a Restricted zone, as follows (see Table 2 to check relationship between parameters and parents):

(5) $$\mathrm{oR}.\mathrm{dep}.\mathrm{day}=\left(\lambda *\left(1-\mathrm{oo}.\mathrm{red}.\mathrm{day}\right)\right)*\inf$$

where λ is the estimated number of oocysts in an oyster (Eq. (1)), “day” is the day of depuration (14, 21, or 30), and “inf” represents the probability of an oocyst remaining viable. We assumed that ‘inf’ follows a beta distribution, involving two shape parameters (a_inf_) and (b_inf_). These parameters were estimated using the total oocyst count and the number of oocysts that remain viable (i.e. number of infective oocysts), while undergoing the depuration process based on unpublished data (J. McClure and S. Greenwood, Personal communication, November 30, 2019) (see Table 2 for detail).

#### 2.1.3 Exposure module

The exposure module estimated the total infective dose (total number of viable oocysts) for the serving size, and estimated oocyst numbers in a single serving (single exposure) of raw oyster meat. Due to the limited availability of raw oyster consumption data in Canada, we used information from a raw oyster consumption survey carried out in Florida, United States (Degner and Petrone, 1994), as a guide to establish consumption patterns of raw oysters in PEI, Canada. Additional information on Canadian seafood consumption was obtained from the following sources: Human Health Risk Assessment Report (Department of Health and Wellness, 2005), Canadian Seafood Survey (Coletto et al., 2011), Farmed Seafood and Canadian Health Report (The Canadian Aquaculture Industry Alliance, 2013), and Statistics Canada (Statistics Canada, 2014).

##### 2.1.3.1 Raw oyster consumption (serving size)

Information from the Florida survey indicated 12 oysters as the most frequent serving size (mean, 13.7 oysters/serving). A risk assessment done in New Brunswick (Canada) by Department of Health and Wellness (2005) established a shellfish serving size of 200 g (not categorised by species). That study, for the estimation of serving size, assumed all shellfish were mussels; however, here, we divided the total amount of shellfish by three to include mussels, oysters, and clams. With the weight of a single oyster between 13 and 16 g (FDA, 2005; WHO and FAO, 2005), we assumed a mean consumption of six oysters per serving and accounted for the variability in serving sizes in the population of interest by using 1, 10, and 30 oysters per serving.

##### 2.1.3.2 Number of *Cryptosporidium* oocysts in a single serving

The shucking of oysters severs both sides of the adductor muscle, a hemolymph organ, causing hemolymph leakage, which affects the ingested dose (Graczyk et al., 2007). The study estimated that approximately 10–32% (mean = 23%) of total viable oocysts reside in the hemolymph (Graczyk et al., 2007). Not all the hemolymph is lost during the shucking process, and it is difficult to estimate the amount lost. We assumed that 23% of oocysts are lost during the handling of oysters prior to consumption, so the ingested dose was multiplied by a factor of 0.77. Finally, ingested dose was estimated as follows for Approved (Eq. (6)), Restricted (Eq. (7)), and Prohibited (Eq. (8)) zones:

(6) oA.cons=oy.cons∗0∗0.77

(7) oR.cons.day=oy.cons∗oR.dep.day∗0.77

(8) oP.cons=oy.cons∗oo.pred∗0.77

where *oy.cons* is the oyster consumption per serving, which takes values of 1, 10, or 30, *oo.pred* is the predicted number of oocysts, “day” is the days of depuration (14, 21, or 30), and 0.77 is the proportion of remaining oocysts after shucking.

##### 2.1.3.3 Dose response model

An exponential model (Messner et al., 2001; Messner and Berger, 2016) was used to estimate the probability of infection after consuming dose “D”. The model assumes that all ingested oocysts are able to initiate an infection (Messner et al., 2001). The parameter “P” represents the fraction of susceptible hosts, while the remainder (1-P) is assumed to be immune. The exponential model assumes that all humans have the same susceptibility to infection, and, therefore P is set to 1 (Messner and Berger, 2016). The parameter “r” defines probability of an oocyst initiating infection in a susceptible host. We adopted a pathogen-specific constant of 0.018 for parameter “r” as suggested by Health Canada (2019) and Messner et al. (2001). This model was compared against additional dose-response data summarized by Messner and Berger (2016), which consisted of data from six experimental challenges of healthy adults exposed to *Cryptosporidium parvum* and *C. hominis* oocysts (Chappell et al., 2006; Messner et al., 2001; Okhuysen et al., 2002).

##### 2.1.3.4 Risk estimation module for infection and illness

This module estimates the probability of infection and illness per serving (i.e. individual risk). The estimated probability of infection per-serving for the Restricted and Prohibited zones was based on the exponential dose-response model. At low Poisson doses, a susceptible host will be infected unless the number of viable oocysts is zero. Infection probability, given Poisson dose “D”, is:

(9) $$P_{\inf}\left(\text{dose}=D\right)=P*\left(1-\exp^{-r*D}\right)$$

The progression from infection to symptomatic illness for *Cryptosporidium* spp. was based on the quantitative risk assessment published by USEPA (2010), where it was assumed that between 20% and 70% of infected individuals would develop symptomatic illness (ill.fr). Hence, we assumed that the probability of illness (P_ill_) had a uniform (0.2, 0.7) distribution.

### 2.2 Model scenarios

Once we developed the core model presented in Fig. 1, a total of 18 scenarios were developed to estimate the per-serving risk of infection and illness and the annual risks for PEI.

*Prohibited zones*: P1, P10, and P30 for consumption of 1, 10, and 30 oysters, respectively.

*Restricted zones*: scenario names were defined as “Zone.Relay-time.Consumption”. Scenarios where 50% of oysters were from 14 relay days and 50% from 21 relay days: R14211, R142110, R142130. Scenarios for oysters from either 14 or 21 relay days: R141, R1410, R1430, R211, R2110, R2130.

*Approved*/*Restricted zones*: it was assumed that 50% of consumed raw oysters were harvested from Approved zones and 50% from Restricted zones: AR141, AR1410, AR1430, AR211, AR2110, AR2130. This assumption is based on the fact that oysters are harvested during two periods. In the first period (Spring: May 1st until July 15th), oysters are harvested in Restricted zones, where it is mandatory to relay them for at least 14 days and provide a microbiological analysis in order to sell them commercially. Alternatively, oyster dealers may relay oysters for a 21-day period and not have to provide a microbiological analysis for commercial sale. During the second harvest period (Fall: August 15th to October 31st), oysters are harvested only in Approved zones with no need for a relay period, and can be sent directly to commercial outlets. It is reasonable to assume that during some periods of the year, commercial-grade oysters will be from the spring season only, the fall season only, or a mix of the two. In addition, Environment Canada indicates that in 2010, 66% of the shellfish growing area, on the Atlantic coast, was designated as Approved zones. Also, a study done in 1975 indicated that about 90% of the oysters were harvested from public beds in PEI; approximately 60% were from beds contaminated by pollution during the spring oyster season, and 40% from the fall season in Approved zones (MacKenzie, 1975). Hence, it is reasonable to assume that 100% or 50% of oysters consumed in a serving come from Restricted zones, depending on the time of year.

Two additional scenarios were built to assess the effect of extended relay days for consumption of 10 and 30 oysters per serving: R3010, and R3030.

### 2.3 Markov-chain Monte Carlo (MCMC) convergence

A stochastic model was developed in OpenBUGS 3.2.3 (Lunn et al., 2009). A total of 80,000 iterations, with a burn-in period of 50,000, were obtained after initializing the model with three chains. The convergence, diagnostic analyses, and summary of all posterior distributions were computed in R, using the CODA package (Plummer et al., 2006). The convergence of the MCMC model was assessed both visually, using the history plots, and formally, using the Brooks-Gelman-Rubin diagnostic (Gelman and Rubin, 1992), which provided an estimate of the shrinkage or scale reduction factor for each of the nodes and scenarios. The distribution of the scale reduction factors (median and 97.5% upper bounds) were plotted to visually assess convergence. Once the model converged, the effective sample size was estimated by running the model for a sufficient number of iterations such that the MCMC error became less than 5% of the posterior standard deviation for monitored nodes. The median and 95% PrI (probability interval) for each stochastic node are reported.

## 3 Results

The number of detected oocysts per oyster was, in general, very low, though a few oysters presented high counts (Table 3, Fig. 2a). Distributions of counts indicated that most of the time oysters were negative, although these Prohibited and Restricted zones could potentially have more than 100 oocysts/oyster. The predicted counts provided a good representation of the observed counts (Table 3; Fig. 2).

**Table 3:** Summary statistics of the observed, true, and predicted number of *Cryptosporidium* oocysts per oyster for the Oyster contamination module. The observed values are estimated from field observations from the Hillsborough River system of Charlottetown (Prince Edward Island, Canada) between July 2011 and July 2012.

Parameter			
	Observed	True	Predicted
Mean	13	23	27
Min	0	0	0
50%	0	0	0
75%	14	25	4
95%	69	125	147
Max	201	363	7890

The regression coefficients from the depuration module are presented in Table 4. We assumed a linear relationship for the predicted number of oocysts at days 14, 21, and 30.

**Table 4:** Regression coefficients from the depuration module of the *Cryptosporidium* spp. risk assessment model obtained with three chains, each with 30,000 iterations, using OpenBUGS. Day is day of measurement of the counts of *Cryptosporidium* oocysts during depuration.

Parameter	Mean	SD	2.5%	50%	97.5%
Day	−0.057	0.002	−0.060	−0.056	−0.051
Intercept	4.434	0.019	4.397	4.434	4.471

**Fig. 3:**
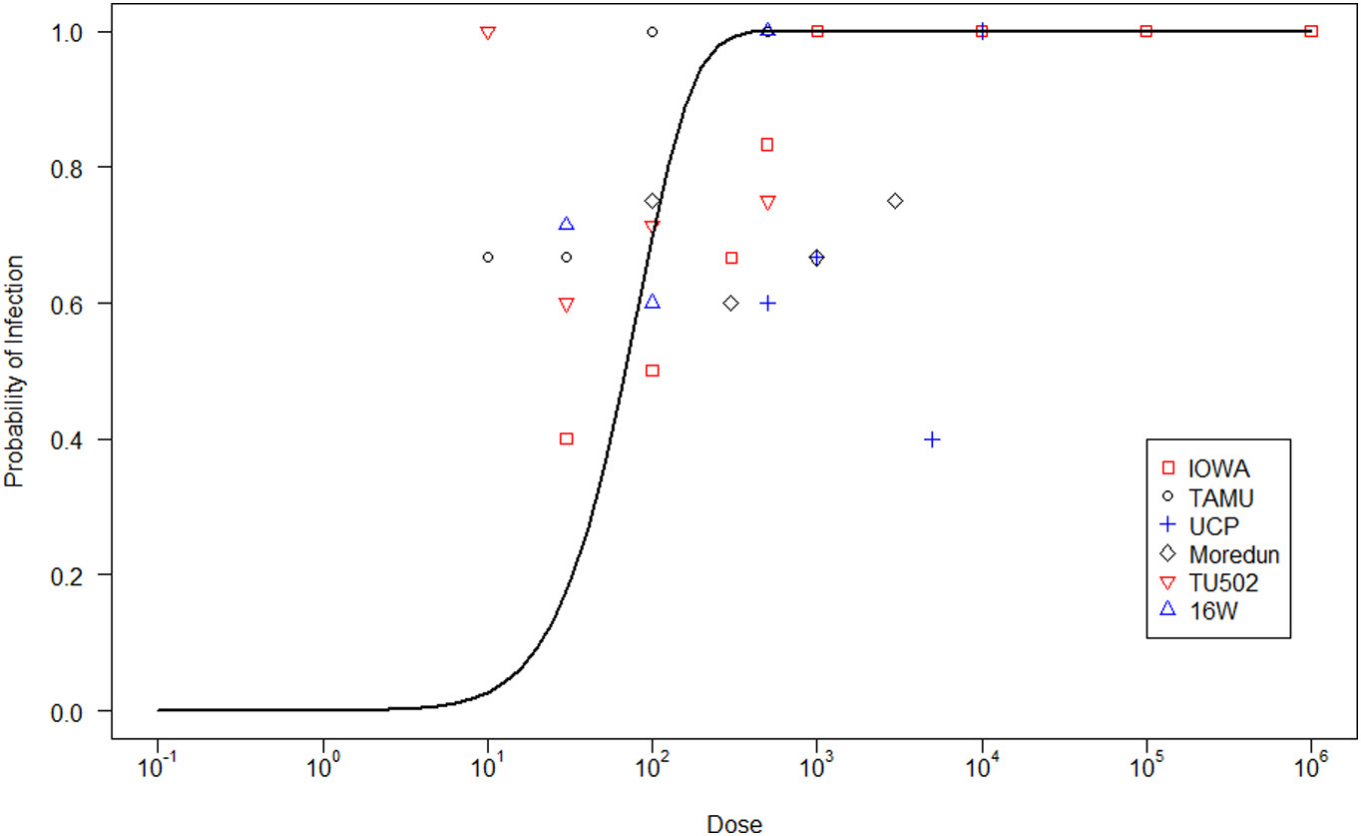
Probabilities of infection by exponential dose-response model, adapted from Messner et al. (2001). This model was compared against the dose-response data summarized by Messner and Berger (2016), but the approach of Health Canada (2019) was used. Legend represents the *Cryptosporidium* strains used in each trial. More details about these data can be found in the cited reference.

Fig. 3 shows the exponential dose-response relationship and the six challenge trials from the literature. This model estimated that the probability of infection reaches a plateau of 1.0 at about 1000 viable oocysts.

**Fig. 4:**
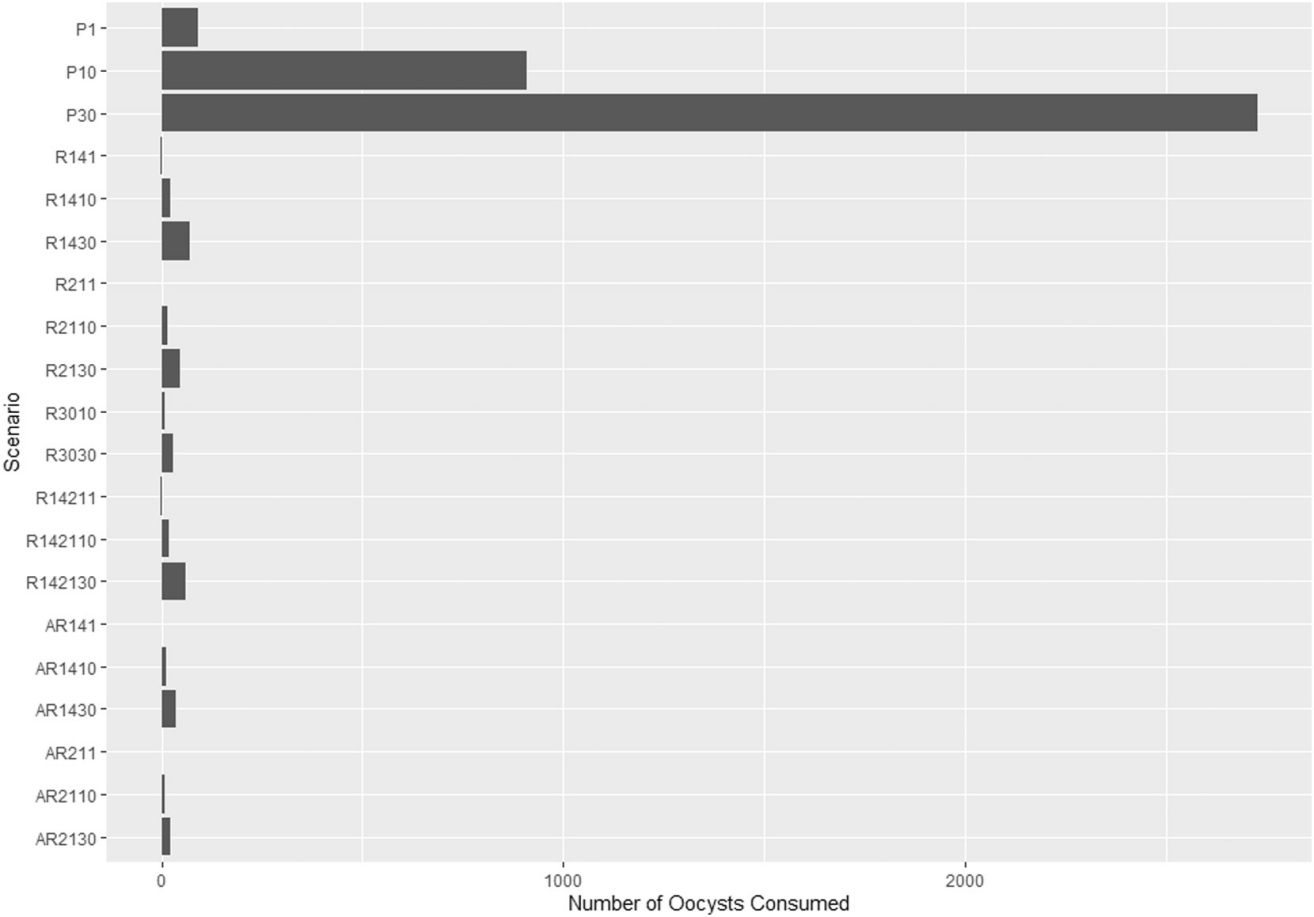
95th percentiles of the predicted number of total *Cryptosporidium* oocysts consumed per oyster serving. Each of the 20 scenarios represents the total dose from the harvesting zone (P = Prohibited, R = Restricted, and A = Approved) with given relay times in Restricted zones and number of oysters consumed per serving. For instance, R142130 = oysters harvested from Restricted zones with relay times between 14 and 21 days and a consumption of 30 oysters in one serving. The median dose for all the scenarios was zero. See Section 2.2 for description of all scenarios.

The size of dose (e.g. oocysts/serving) consumed depended on the harvesting zone, relay time, and number of oysters per serving (Table 5; Fig. 4). Most of the time the dose consumed was zero (Table 5), since there was a high probability of zero counts of oocysts/oyster (Fig. 2).

**Table 5:** Predicted 50th, 75th, and 95th percentiles of the number of oocysts consumed per oyster serving by scenario from the *Cryptosporidium* spp. risk assessment model obtained with three chains, each with 30,000 iterations, using OpenBUGS.

Name	Scenario^a^			Percentile		
	Zone	Relay time	Consumption	50th	75th	95th
P1	Prohibited	–	1	0	2	90
P10			10	0	23	909
P30			30	0	69	2726
R141	Restricted	14	1	0	0	2
R1410			10	0	1	24
R1430			30	0	2	72
R211		21	1	0	0	2
R2110			10	0	0	16
R2130			30	0	1	48
R3010		30	10	0	0	10
R3030			30	0	1	29
R14211		14–21	1	0	0	2
R142110			10	0	1	20
R142130			30	0	2	60
AR141	Approved - restricted		1	0	0	1
AR1410		14	10	0	0	12
AR1430			30	0	1	36
AR211			1	0	0	1
AR2110		21	10	0	0	8
AR2130			30	0	1	24

a See full description of each scenario in Section 2.2.

**Fig. 5:**
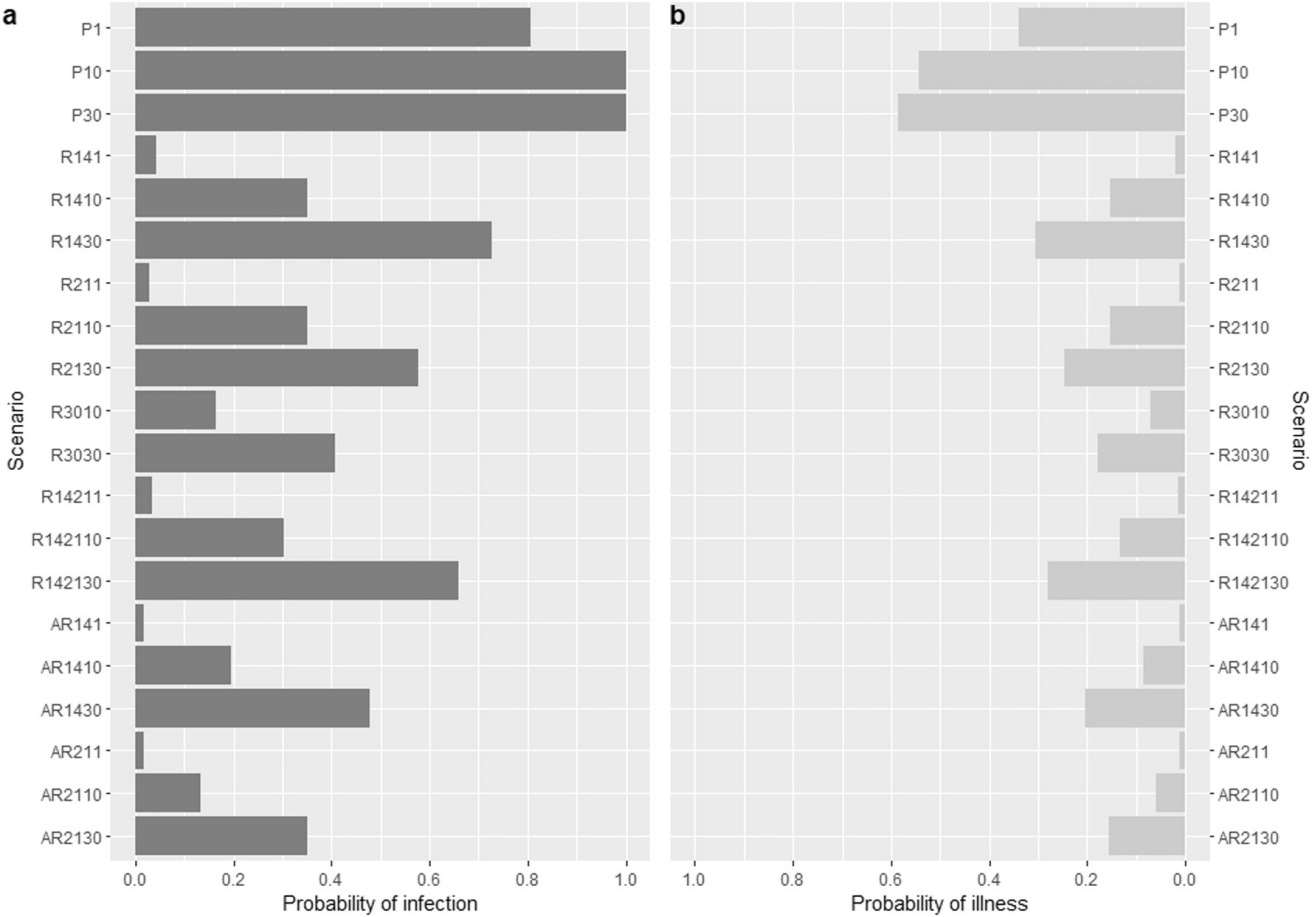
95th percentiles of probabilities of infection and illness from raw oyster consumption in Prince Edward Island. Each of the 20 scenarios represents the probability from the harvesting zone (P = Prohibited, R = Restricted, and A = Approved) with given relay times in Restricted zones and number of oysters consumed per serving. For instance, R142130 = oysters harvested from Restricted zones with relay times between 14 and 21 days and a consumption of 30 oysters in one serving. See Section 2.2 for description of all scenarios.

The median per-serving probabilities of infection and illness for all the scenarios were equal to zero. However, when assessing the 95th percentiles of these probabilities, we found the greatest reduction when the relay time was extended to 30 days and an individual consumed, at most, 10 oysters in one serving. 95th percentiles were over 0.35 and 0.15 for the probabilities of infection and illness, respectively, for all scenarios when 30 oysters per serving were consumed (Fig. 5).

Comparing the probabilities at different relay times (14 vs 30 days) for the Restricted zone, the probability of infection reduced from 0.35 to 0.16, while probability of illness changed from 0.15 to 0.07, when 10 raw oysters were consumed in one serving. This suggested a reduction of 53% for both probabilities of infection and illness, when the relay time was extended from 14 to 30 days.

Model diagnostics included the history of the chains, and Brooks-Gelman-Rubin plots, and showed no evidence that convergence had not occurred after obtaining samples from 30,000 iterations.

## 4 Discussion

The present risk assessment, together with prior studies on *Cryptosporidium* spp. in raw oysters for human consumption, demonstrates that oysters can be contaminated with *Cryptosporidium* spp. and information on complete elimination of this pathogen is currently unavailable (Dawson, 2005). However, the likely overall risk per serving and per year remains negligible under the current conditions in PEI. Although the results from this model indicate that the risk of illness could be very high in the area with high degrees of contamination (i.e. Prohibited zones), the likelihood of occurrence is very low (e.g. 95th percentile). The data from this study were obtained from only one harvesting area in PEI, Hillsborough River. The area has higher urban density than other regions (e.g., Malpeque Bay) and the pollution control plant for Charlottetown is located on the river. Although water has been treated and undergone the UV inactivation process before emptying into the river (City of Charlottetown, n.d.), some oocysts may not be removed in these treatments. The plant may occasionally pump or leak untreated sewage into the river, thus increasing the risk of *Cryptosporidium* contamination.

The results from the scenarios assessed in this model could represent future changes in oyster contamination due to factors associated with climate change (e.g., increase in frequency and intensity of precipitation). The changes in the susceptible population (e.g., increased numbers of elderly or immunocompromised individuals) could lead to more illness following the contamination. If so, we speculate that depuration periods established by governments are not long enough to ensure pathogen elimination. Gómez-Couso et al. (2003) also found that a depuration time of 14 days was not enough to remove all oocysts present. Our model predicted that oysters harvested from Restricted zones and relayed for 14 days could have enough oocysts to initiate an infection. In addition, oocysts can remain viable in harvested waters for over a year (Tamburrini and Pozio, 1999), which suggests the need for implementation of strategies to prevent or reduce *Cryptosporidium* contamination in edible molluscan shellfish (Gómez-Couso et al., 2003, Gómez-Couso et al., 2004). However, the lack of evidence of human cryptosporidiosis linked to oysters generates a great deal of scepticism about the epidemiological importance of scientific reports and the necessity of reducing or eliminating the presence of waterborne *Cryptosporidium* in oyster harvesting waters (Graczyk et al., 2006; Willis et al., 2014).

The 14-day relay period established by Canadian authorities for harvesting in Restricted zones seems prudent, though insufficient, as this relay period has been shown to be enough to eliminate fecal coliforms but not *Cryptosporidium* oocysts, which can remain viable in the oyster for over a month (Gómez-Couso et al., 2003; Schijven et al., 2013; Sutthikornchai et al., 2016). The effectiveness of the depuration process will depend, in addition to other factors, on the number of parasites in estuaries, which could be correlated with the presence of agricultural land in the surrounding areas and the intensities of rain events (Aguirre et al., 2016). Oysters filter large volumes of water to feed, and during this process they accumulate toxins, contaminants, and microorganisms, such as *Cryptosporidium* oocysts, in their tissues (Iwamoto et al., 2010; Willis et al., 2013b). Heavy rain events can increase agricultural and urban runoff, while wastewater effluents intensify due to steady growth of the human population.

Findings from the present study could be used to inform decisions about control measures of not only the loads of fecal coliforms in harvest waters but also *Cryptosporidium* spp., and confirm previous findings that the standard water quality parameters, such as fecal indicator coliform counts, do not correlate with *Cryptosporidium* oocyst contamination in edible oysters (Freire-Santos et al., 2000; Gómez-Couso et al., 2003, Gómez-Couso et al., 2004; Graczyk et al., 2006). This is of particular concern when foodborne illnesses following consumption of raw oysters occur, even when fecal coliform testing of shellfish harvesting waters demonstrates compliance with the National Shellfish Sanitation Program criteria and sanitation at the oyster harvesting facilities meets standards set by regulatory authorities (Graczyk et al., 2006; Graczyk and Schwab, 2000).

We could estimate the annual exposure using the number of raw oysters served in a year. In 2012, the total oyster landings in PEI was 2,786,543 kg (unshucked) (PEI Department of Agriculture and Fisheries, n.d.). Assuming that, on average, an unshucked oyster weighs 60 g (unpublished) and this is about one-half of the shelled weight, then approximately 23,221,191 oysters are harvested per year in PEI. Economic information from Aquaculture Alliance PEI and expert judgments support the assumption that 70% of the total landings produced in PEI comes from fisheries and 5% of that is consumed locally either cooked or raw (industry expert). Since oysters from fisheries are more likely to be of low quality and more likely to be cooked, we assume that only 15% of these oysters are consumed raw, and, therefore an estimated total of 121,911 oysters will be consumed raw in PEI during one year.

A study on PEI revealed that of 658 human fecal samples without IGI-specific symptoms, 22% were positive for *Cryptosporidium* spp., of which 28% and 72% were identified as *C. hominis* and *C. parvum*, respectively (Budu-Amoako et al., 2012). Approximately 40% of the positive cases were found in people between 20 and 65 years of age (Budu-Amoako et al., 2011), the age range where the consumption of oysters is most frequent. However, there have been no reports of *Cryptosporidium* or cryptosporidiosis associated with consumption of oysters in PEI. If changing climatic conditions increase the contamination levels of oysters, then there is a potentially high risk of infection and illness, but this is very difficult to validate. Not every individual with IGI will seek medical care; not everyone who seeks medical care will be asked to provide a stool sample; not all stool submissions will be tested for *Cryptosporidium* oocysts; not all of these will test positive; not all positive samples will be reported; and so on (MacDougall et al., 2008; Majowicz et al., 2005). If an IGI case is determined to be caused by *Cryptosporidium* spp., the ability of physicians to correlate that exposure to the consumption of shellfish or another food item is extremely limited (Graczyk et al., 2005). The incubation period for *C. parvum* ranges from one to 12 days (average, 7 days) (Heymann, 2008), which may contribute to the lack of reported human cases, as individuals do not display visible symptoms immediately after consumption. And, food consumption histories obtained are typically focused on the few days before onset of diarrhea and become unreliable after 2–3 days.

Despite these limitations, the Bayesian modelling approach used in this study allowed us to combine different sources of information into a single model framework and integrate them into different modules, while propagating uncertainty until the final risk estimates. In the present study, we used experimental information (depuration module), field information (contamination module), and literature (dose-response model), and linked them into a single model framework. The advantage of this approach is that it allows users to trace back to a particular scenario from the final outcome and identify the values of particular input information. Moreover, the Bayesian approach allows additional temporal and regional data to be incorporated in the assessment to provide updated risk estimates, and this approach likely has utility for assessing risk of other waterborne parasites with similar modes of transmission (e.g. *Giardia* spp.).

## 5 Limitations and data gaps

The results of this model are based on a set of assumptions and information needed to develop each of the modules of the assessment. The estimated risk from our study could have been overestimated, given that the level of contamination with *Cryptosporidium* spp. in oysters from other harvesting areas in PEI might be much lower than the loads found in the Hillsborough River system of Charlottetown. Since genotyping information for the oocysts found in the oysters was not available for this study, we have assumed that all the oocysts present in the oyster are human-pathogenic and, therefore, will infect humans (e.g. zoonotics). It has been shown that approximately 5% of the total oocysts present in water samples in Ontario were pathogenic (Lapen et al., 2016), while prevalence estimates between 16% and 89% of pathogenic oocysts have been reported in oysters in eastern USA and Canada (Willis et al., 2013b). Future monitoring of *Cryptosporidium* spp. in oysters should include at least some off-the-slide genotyping to obtain more information on the proportion of human-pathogenic species.

In addition, the lack of information on cases of cryptosporidiosis related to the consumption of the raw oysters makes validation of the model difficult. For instance, the model estimates ignore the tourist population (cruise ships and short-term summer visitors) present during the harvesting season, who may preferentially consume a dozen raw oysters in two restaurant meals. Many of them will stay for less than the incubation period and, hence, would have an IGI reporting likelihood close to zero.

The major limitation with dose-response models of this kind is the lack of data at low doses; for *Cryptosporidium* this corresponds to doses of less than 10 oocysts. Low-dose trials might reveal another dose-response model as a better fit, for instance the beta-Poisson model (Messner and Berger, 2016).

Validation of the overall risk estimates (annual risk per total exposure to the total oysters consumed in PEI) requires detailed data on the number of illnesses associated with consumption of raw oysters consumed from PEI landings. Unfortunately, there are no survey data associating oyster consumption with cryptosporidiosis in PEI. More precise information about cases of cryptosporidiosis would need to be collected in human health registries to properly validate this model.

The length of the depuration period is another major assumption used in this model, since data are from experimental studies with durations interpolated using regression models. The magnitude and direction of the relay effect at longer durations are difficult to predict, and field studies should be conducted to assess this relationship. However, information from the literature was used to verify that the percentage reduction predicted from our model at longer periods could be expected. For this assessment, we assumed that similar *Cryptosporidium* contamination in oysters, as observed for the Hillsborough River system of Charlottetown between July 2011 and July 2012, is representative of all harvesting zones across PEI.

Data from other locations on the island could be collected and incorporated into this model to increase the external validity of the results.

## 6 Conclusions

This study found that the median probability of infection and developing cryptosporidiosis (e.g. illness) due to the consumption of raw oysters in PEI was zero. However, there might be situations where the risk, as represented by these probabilities, could be substantial (e.g. 75th–95th percentile of all scenarios). At present, *Cryptosporidium* spp. loads in water or shellfish do not have to meet any regulatory criteria, but findings from this study should stimulate debate concerning the need for such guidelines. The scenario analyses provided information about infection risks for specific concentrations of *Cryptosporidium* oocysts in their source oysters. Extending relay periods of 14 and 21 days for oysters harvested in Restricted zones to 30 days showed a decrease of over 50% in both the probabilities of infection and illness, but these estimates were made from experimental data. A field study should be conducted to validate this finding.

Overall, we believe that our approach provides valuable information because it creates awareness about the effects of current relay times of pathogens of public health importance.

## Declaration of competing interest

The authors declare they have no competing financial interests.
